# Pain Beliefs, Coping Approaches, and Related Factors in Nursing Students

**DOI:** 10.7759/cureus.60454

**Published:** 2024-05-16

**Authors:** Seçkin Karakuş, Serdar Sarıtaş

**Affiliations:** 1 Nursing, Erzincan Binali Yildirim University, Erzincan, TUR; 2 Medical Biology, Malatya Turgut Ozal University, Malatya, TUR

**Keywords:** pain management, pain coping, pain beliefs, pain, nursing students

## Abstract

Objectives

Pain, a common human experience, is also experienced by nursing students, and pain beliefs, thoughts, and behaviors toward pain play an important role in coping with pain. There is insufficient data about the relationship between pain beliefs and pain coping strategies. Thus, this study aims to reveal the relationship between pain beliefs and pain coping approaches of nursing students and affecting factors.

Methods

A descriptive, cross-sectional, and correlational design was used, and the data were collected with respondent characteristics form, the Numerical Rating Scale (NRS) and the Pain Beliefs Questionnaire (PBQ), by researchers from 380 nursing students in the nursing department.

Results

Nursing students who used non-pharmacological interventions to cope with pain had higher levels of psychological belief (PBQ-P) scores (4.97±0.86) than organic belief (PBQ-O) scores (3.90±0.71) and the difference was statistically significant (p<0.001). According to the multivariate linear regression analysis results, nursing students' gender, utilizing non-pharmacological interventions, and NRS scores affected PBQ-P scores by 87.1% (R^2^=0.871) and PBQ-O scores by 81.0% (R^2^=0.810).

Conclusions

As can be seen from the results of this study, the higher psychological beliefs of nursing students who use non-pharmacological interventions to cope with pain are an example of this situation. In light of the information in this study, it should be taken into consideration that both psychological and organic beliefs have a strong relationship with pain intensity and pain coping approaches. Nursing students, the nurses of tomorrow, should be aware of the impact of psychological and organic beliefs on individuals' pain experiences and coping approaches and should take this into account when planning nursing care.

## Introduction

Individuals' pain beliefs, perceptions, and attitudes toward pain play an important role in experiencing and adapting to pain [1]. Although pain is a subjective phenomenon, attitudes and beliefs toward pain are derived from social interactions and must be evaluated within the cultural context in which they developed. Ultimately, these attitudes and beliefs can be potential barriers to effective pain management [2]. Pain beliefs, which are stated to affect individuals' ability to cope with pain effectively, the course of the treatment process, and their behavior and attitudes toward pain are affected by factors such as gender, age, presence of physical limitations, and depression status [3,4].

Pain beliefs, which determine how people cope and manage when experiencing pain, are shaped by an individual's personal views on pain management [3]. Pain, a common human experience, is also experienced by nursing students, and different approaches are used to cope [2]. In studies conducted with nursing students, it is determined that pain beliefs are especially affected by gender, undergraduate education year, the idea of who has control of pain, methods used to cope with pain, and pain intensity [5-9]. In studies conducted to evaluate individuals' pain beliefs and coping strategies, it is stated that whether the pain beliefs of individuals have a psychological or organic origin may cause differences in the pain coping strategy and treatment process [4,10,11]. In a study conducted by Işık and Yanık in 2022 to examine the cultural perceptions of nursing students regarding pain and the methods used in pain treatment, it was reported that nursing students who applied traditional cultural methods to cope with pain had higher psychological beliefs (Pain Beliefs Questionnaire-psychological beliefs subscale score (PBQ-P)) scores [7]. The aim of this study is to reveal the relationship between pain beliefs and pain coping approaches of nursing students and affecting factors.

## Materials and methods

A descriptive, cross-sectional, and correlational design was used in this study. The data were collected via face-to-face method using a survey. The sample consisted of 380 of the 472 students in Erzincan Binali Yildirim University, Health Sciences Nursing Department, Turkey, between January 2020 and April 2020. All students who met the inclusion criteria for the study were included in the research without going to the sample selection method. The inclusion criteria were age 18 or older, having no communication problems, and not experiencing chronic pain. Written approval was obtained from the Erzincan Binali Yildirim University Human Research Ethics Committee (Decision No: 25/12/2019-12/16) and Erzincan Binali Yildirim University Faculty of Health Sciences (Decision No: 11327278-210.01-E.59180).

Data were collected with respondent characteristics form, the Numerical Rating Scale (NRS) and the Pain Beliefs Questionnaire (PBQ), by the researcher via the face-to-face method using a survey. After the students were informed about the study, their consent was obtained from people who agreed to participate. Participants completed their survey within 10 minutes.

The respondent characteristics form was prepared based on the literature review conducted by the researchers and consists of 10 questions [5,9,12]. The respondent characteristics form include gender, age, undergraduate education year, and income status, among others.

NRS is a one-dimensional numerical assessment tool used to determine the intensity of pain. The measurement is made by specifying a number from “0: no pain” to “10: worst possible pain.” It allows more accurate expression of pain and easy recording of data [13].

PBQ was developed by Edwards et al. in 1992 to assess beliefs about the cause and treatment of pain. The 12-item scale has organic beliefs (total score of one to six) and psychological beliefs (total score of one to six) subscales. Higher scores indicate higher pain beliefs [14]. Turkish validity and reliability study of the scale was conducted by Sertel Berk in 2006. Sertel Berk reported the Cronbach’s Alpha of the Turkish version of the PBQ “psychological beliefs” and “organic beliefs” subscale scores as 0.73 and 0.71, respectively, which were 0.73 and 0.61 in this study, respectively [3].

The data were analyzed using the SPSS Statistics 22.0 (Statistical Package for Social Sciences) at a significance level of 0.05. The categorizable data of the research are expressed as numbers (n) and percentages (%), and the numerical data are expressed as mean (X̄) ± standard deviation (SD). Mann-Whitney U test was used to compare two independent groups. ANOVA was used to compare more than two independent groups, and in cases where the analysis was significant, the Tukey post hoc test was used to determine the source of differences. The factors affecting PBQ-P and PBQ-O scores were analyzed using multivariate linear regression.

## Results

The mean age of the nursing students was 20.39±1.58 years, 271 (71.3%) were female, 106 (27.9%) are first-year students, 220 (57.9%) have income equal to their expenses, 233 (61.3%) believe pain is in their own control, 239 (62.9%) describe the pain threshold as moderate, 183 (48.2%) first wait for the pain to subside when experiencing pain and take painkillers if it does not, and 265 (69.7%) benefit from non-pharmacological interventions in coping with pain (Table 1).

**Table 1:** Respondent characteristics (total, n: 380) X̅±SD, mean ± standard deviation

Variables	Category	Total, n: 380 (%)
Age (year)	X̅±SD	20.39±1.58 (Min: 18; Max: 29)
Gender	Female	271 (71.3)
	Male	109 (28.7)
Undergraduate education year	1st year	106 (27.9)
	2nd year	105 (27.6)
	3rd year	95 (25.0)
	4th year	74 (19.5)
Income status	Income is more than expenses	51 (13.4)
	Income equals to expenses	220 (57.9)
	Income is less than expenses	109 (28.7)
Pain control is in	Self	233 (61.3)
	God	116 (30.5)
	Physician	30 (7.9)
	Nurse	1 (0.3)
When experiencing pain	I just wait for the pain to go away and do nothing else.	52 (13.7)
	First, I wait for the pain to go away, if not, I take a painkiller.	183 (48.2)
	I take painkillers immediately.	29 (7.6)
	First, I wait for the pain to go away, and if it does not, I resort to a non-pharmacological pain-relieving method.	99 (26.1)
	I immediately resort to a non-pharmacological pain-relieving method.	17 (4.5)
Utilizing non-pharmacological interventions	Yes	265 (69.7)
	No	115 (30.3)

**Figure 1:**
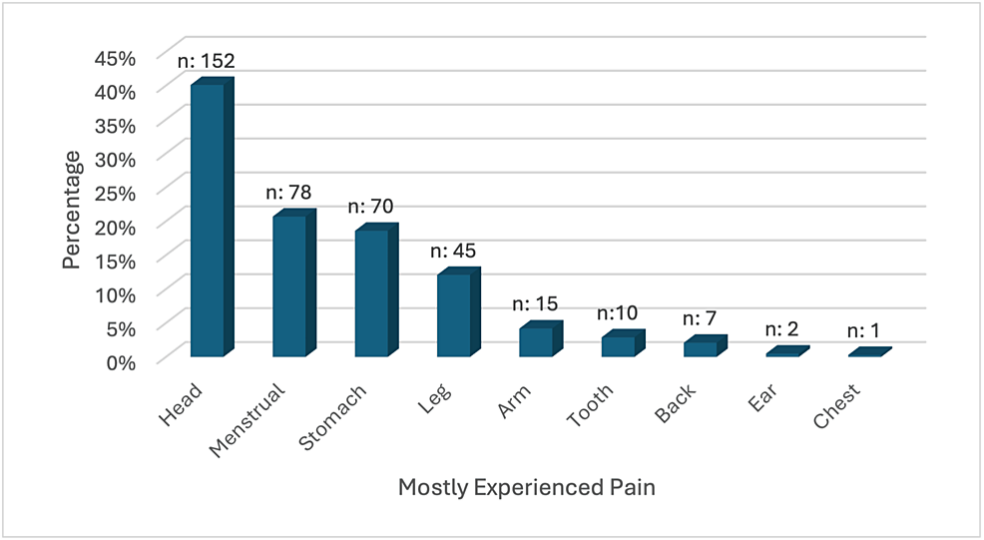
Nursing students mostly experience pain One option was selected.

Among nursing students, 152 (40.1%) experience headache, 78 (20.6%) experience menstrual pain, 70 (18.4%) experience abdominal pain, and 45 (11.8%) experience leg pain mostly (Figure 1).

**Figure 2:**
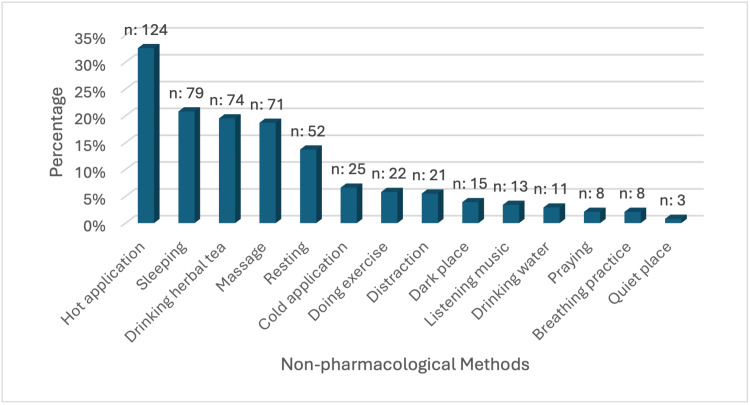
Non-pharmacological methods mostly used by nursing students to cope with pain More than one option was selected.

In pain coping with non-pharmacological methods, 124 (32.6%) of the nursing students use the hot application, 79 (20.8%) prefer sleeping, 74 (19.5%) drink herbal tea, 71 (18.7%) benefit from massage, and 52 (13.7%) prefer resting (Figure 2).

The female nursing students' mean psychological belief (PBQ-P) scores (4.97±0.86) and mean NRS scores (7.44±1.17) were found to be statistically significant (p<0.05, Table 2). The average PBQ-P scores (5.13±0.75) and average NRS scores (7.67±1.12) of the nursing students in the third year of undergraduate education were found to be statistically significant compared to the nursing students in the first year and second year (p<0.05, Table 2). The average PBQ-P scores (5.04±0.84) of the nursing students who defined their income as less than their expenses were found to be statistically significant compared to the nursing students who defined their income as more than their expenses (p<0.05, Table 2). The average organic belief (PBQ-O) scores (4.01±0.74) of the nursing students who stated pain is controlled by God were found to be statistically significant compared to the nursing students who stated pain is controlled by the individual (p<0.05, Table 2). The average PBQ-P scores of the nursing students who defined their pain threshold as high (4.88±0.97) and medium (4.92±0.85) were found to be statistically significant compared to the nursing students who defined their pain threshold as low (p<0.05, Table 2). The mean NRS score (7.45±1.13) of the nursing students who defined their pain threshold as medium was found to be statistically significant compared to the nursing students who defined their pain threshold as low (p<0.05, Table 2). The average PBQ-P scores of the nursing students, when experiencing pain, who first waited for the pain to go away and if it did not, resorted to a pain-relieving method other than medication (5.10±0.81) was found to be statistically significant compared to the nursing students who just waited for the pain to go away and did nothing else (4.68±1.03), who first waited for the pain to go away and then took painkillers if it did not go away (4.80±0.90), and who took painkillers immediately (4.69±0.96) (p<0.05, Table 2). The average PBQ-P scores (4.97±0.86) and average NRS scores (7.46±1.20) of the nursing students who used non-pharmacological interventions to cope with pain were found to be statistically significant (p<0.05, Table 2). The average PBQ-P scores of nursing students who use drinking water and breathing practice to cope with pain, the average PBQ-O scores of nursing students who benefit from praying, and the average NRS scores of nursing students who benefit from cold application are the highest (Table 2).

**Table 2:** Relationship between respondent characteristics, PBQ subscales, and NRS X̅±SD, mean ± standard deviation; PBQ-P, Pain Beliefs Questionnaire-psychological beliefs subscale score; PBQ-O, Pain Beliefs Questionnaire-organic beliefs subscale score; NRS, Numerical Rating Scale

Variables	Category	PBQ-P	PBQ-O	NRS
		X̅±SD	X̅±SD	X̅±SD
Gender	Female	4.97±0.86	3.88±0.67	7.44±1.17
	Male	4.60±0.98	3.92±0.81	7.14±1.35
	p-value	<0.001	0.677	0.032
Undergraduate education year	1st year	4.73±0.97	3.86±0.71	7.22±1.30
	2nd year	4.70±0.95	3.86±0.76	7.20±1.29
	3rd year	5.13±0.75	4.00±0.69	7.67±1.12
	4th year	4.92±0.87	3.84±0.69	7.36±1.12
	p-value	0.003	0.405	0.030
	Post-hoc	3>1-2		3>1-2
Income status	Income is more than expenses	4.58±0.93	3.92±.066	7.09±1.20
	Income equals to expenses	4.84±0.92	3.89±0.71	7.34±1.24
	Income is less than expenses	5.04±0.84	3.88±0.75	7.52±1.21
	p-value	0.010	0.962	0.120
	Post-hoc	3>1		
Pain control is in	Self	4.93±0.90	3.79±0.66	7.33±1.19
	God	4.71±0.93	4.01±0.74	7.31±1.28
	Physician	4.96±0.90	4.23±0.84	7.80±1.37
	Nurse	4.00±0.00	3.80±0.00	-(n=1)
	p-value	0.123	0.004	0.241
	Post-hoc		2>1	
When experiencing pain	I just wait for the pain to go away and do nothing else.	4.68±1.03	3.88±0.72	7.13±1.25
	First, I wait for the pain to go away, if not, I take a painkiller.	4.80±0.90	3.90±0.73	7.31±1.25
	I take painkillers immediately.	4.69±0.96	4.09±0.62	7.34±1.28
	First, I wait for the pain to go away, and if it does not, I resort to a non-pharmacological pain-relieving method.	5.10±0.81	3.90±0.65	7.62±1.14
	I immediately resort to a non-pharmacological pain-relieving method.	4.95±0.86	3.49±0.89	7.05±1.24
	p-value	0.040	0.159	0.106
	Post-hoc	4>1-2-3		
Utilizing non-pharmacological interventions	Yes	4.97±0.86	3.90±0.71	7.46±1.20
	No	4.62±0.98	3.86±0.73	7.13±1.28
	p-value	0.001	0.587	0.017
Non-pharmacological interventions	Hot application	5.02±0.79	3.86±0.68	7.46±1.08
	Sleeping	5.16±0.79	3.95±0.67	7.67±1.12
	Drinking herbal tea	4.93±1.00	3.86±0.73	7.40±1.37
	Massage	4.84±0.89	3.84±0.76	7.29±1.17
	Resting	5.19±0.69	3.98±0.68	7.75±0.98
	Cold application	5.16±0.95	4.09±0.87	7.80±1.38
	Doing exercise	5.05±0.81	3.95±0.61	7.54±0.85
	Distraction	5.19±0.75	3.97±0.79	7.71±1.14
	Dark place	5.23±0.69	3.86±0.61	7.60±0.91
	Listening music	5.24±0.75	3.78±1.19	7.46±1.33
	Drinking water	5.32±0.86	3.70±0.50	7.54±1.03
	Praying	4.90±0.88	4.12±0.93	7.62±1.30
	Breathing practice	5.32±0.59	3.61±0.60	7.37±0.91
	Quiet place	5.10±0.36	3.53±0.25	7.33±0.57

The effects of gender, utilizing non-pharmacological interventions, and NRS scores on PBQ-P and PBQ-O scores were tested using a multivariate linear regression model. Regression analysis results showed that all these variables had a significant effect on the model. According to the analysis results, nursing students' gender, utilizing non-pharmacological interventions, and NRS scores affected PBQ-P scores by 87.1% (R^2^=0.871) and PBQ-O scores by 81.0% (R^2^=0.810, Table 3).

**Table 3:** Multivariate linear regression model for the PBQ subscale scores PBQ-P, Pain Beliefs Questionnaire-psychological beliefs subscale score; PBQ-O, Pain Beliefs Questionnaire-organic beliefs subscale score; NRS, Numerical Rating Scale

Subscales	Unstandardized coefficients β	Standardized coefficients β	t-value	p-value
PBQ-P				
Constant	162		1110	268
NRS	628	850	33205	<0.001
Gender	156	78	3010	0.003
Non-pharmacological interventions	-115	-58	-2238	0.026
PBQ-O				
Constant	481		3516	<0.001
NRS	475	819	26759	<0.001
Gender	-162	-102	-3318	0.001
Non-pharmacological interventions	87	56	1806	72

## Discussion

This study investigated the effects of nursing students' respondent characteristics, pain beliefs, and pain intensity on pain-coping approaches. Nearly half (40.1%) of the nursing students experience headaches, whereas one-fifth experience menstrual pain and stomachache (Table 1). In a study, nursing students experienced headaches (56.7%), stomachache (26.6%), waist pain (25.7%), and back pain (24.5%) [7]. In another study, nursing students experienced headaches (53.3%), abdominal pain (42.4%), and waist pain (33%) [15]. The most common pain experiences of the nursing students in this study are in line with the current literature.

The PBQ is commonly utilized to evaluate individuals’ pain beliefs. In this study, females’ psychological beliefs scores were significantly higher than males' (p<0.05), and no significant difference was found in the organic beliefs point average. In a study similar to this study, females' psychological beliefs scores were significantly higher than males' (p<0.05), and no significant difference was found in the organic beliefs point average [5]. In another study similar to this study, females' psychological beliefs scores were significantly higher than males (p<0.05) whereas males' organic beliefs scores were significantly higher than females (p<0.05) [7]. Unlike this study, no significant difference was found in average PBQ-P scores but female’s average PBQ-O scores are higher than males (p<0.05) [6]. According to these results, it can be said that females think that pain is caused by psychological reasons such as stress and anxiety, while males think that it is caused by organic reasons such as injury and damage generally.

NRS is a short assessment tool commonly used to determine pain intensity. Studies examining the relationship between gender and pain intensity of nursing students are limited. When we consider pain intensity from a gender perspective, females’ NRS scores were significantly higher than males (p<0.05) in this study. In a study conducted with individuals with chronic low back pain, it was determined that females' NRS scores were higher and there were significant differences between gender groups (p<0.001) in terms of psychological pain beliefs and pain intensity [16]. Epidemiological studies indicate that females experience more pain, react more negatively to pain, and have a lower pain threshold compared to males. Studies examining the role of gender expectations on pain suggest that gender differences in gender expectations may influence pain expression [17,18]. According to these results, it can be said that the average experienced pain intensity is higher in females than in males because females experience more frequent and severe pain in their daily lives and are less likely to avoid expressing their pain.

When examining how undergraduate education year affects pain beliefs, in this study, the mean score of 3rd year nursing students was found to be higher than that of 1st and 2nd year nursing students in the psychological beliefs scores (p<0.05). In a study similar to this study, it was determined that the organic beliefs scores of the 3rd year nursing students were higher than the 1st grade nursing students (p<0.05), and the psychological beliefs scores were higher than the students in all other years (p<0.05) [9]. In another study, it was found that the organic beliefs scores of 4th year nursing students were significantly lower than all other years (p<0.001) [6]. It is thought that this is due to the fact that as nursing students' theoretical and clinical experiences increase, they associate pain with psychological causes and their coping strategies with pain change. Studies examining the relationship between income status and pain beliefs of nursing students are limited. When examining how income status affects pain beliefs, in this study, the average PBQ-P scores of the nursing students who defined their income as less than their expenses were found to be higher than those who defined their income as more than their expenses (p<0.05). In a study conducted with total knee and hip replacement surgery patients, it was found that patients with low-income status had higher organic and psychological pain belief scores (p<0.05) [19]. In another study conducted with adults, low-income status was associated with higher psychological beliefs scores (p<0.05) [20]. It is thought that this situation arises from the widespread belief that pain is caused by psychological factors such as socioeconomic stress, as individuals with low-income levels experience financial difficulties. When examining how the idea of who has control of pain affects pain beliefs, in this study, the average PBQ-O scores of the nursing students who stated pain is controlled by God were found to be statistically significant compared to the nursing students who stated pain is controlled by the individual (p<0.05). In a study similar to this study, the average PBQ-O scores of nursing students who thought pain control was in God were found to be significantly higher (p<0.05) [5]. In another study, the average PBQ-O scores of nursing students who thought they needed to see a doctor to manage pain were found to be statistically significant (p<0.05) [7]. It is thought that nursing students with higher PBQ-O scores believe pain is caused by a situation that refers to organic beliefs such as an injury or disease, therefore, rely on external sources such as God or doctor rather than themselves in controlling the pain. When examining how pain beliefs affect what is done while experiencing pain, in this study, the average PBQ-P scores of the nursing students, when experiencing pain, who first waited for the pain to go away and if it did not, resorted to a pain-relieving method other than medication was found to be statistically significant compared to the nursing students who just waited for the pain to go away and did nothing else, who first waited for the pain to go away and then took painkillers if it did not go away, and who took painkillers immediately (p<0.05). In a study, the average PBQ-O scores of the nursing students, when experiencing pain, who took painkillers immediately were found to be statistically significant compared to the nursing students who first waited for the pain to go away and then took painkillers if it did not go away and who immediately resort to a non-pharmacological pain relieving method (p<0.05) [9]. This situation is thought to be due to the fact that nursing students with higher PBQ-P scores turn to non-pharmacological interventions that refer to psychological beliefs in coping with pain, and nursing students with higher PBQ-O scores turn to pharmacological methods that refer to organic beliefs in coping with pain. When examining how pain beliefs affect utilizing non-pharmacological interventions, in this study, the average PBQ-P scores of the nursing students who used non-pharmacological interventions to cope with pain were found to be statistically significant (p<0.05). In a study similar to this study, the average PBQ-P scores of the nursing students who applied traditional cultural methods to cope with pain were found to be statistically significant (p<0.05) [7]. In another study similar to this study, the average PBQ-P scores of the nursing students using nonpharmacological methods in their pain control were found to be statistically significant (p<0.05) [21]. In a different study, the average PBQ-P and PBQ-O scores of the nursing students who applied traditional cultural methods to cope with pain were found to be statistically significant (p<0.05) [6]. This is thought to be due to the fact that nursing students with higher PBQ-P scores find non-pharmacological interventions that refer to psychological beliefs effective in coping with pain. In this study, almost a third of the nursing students use the hot application method, whereas one-fifth benefit from sleeping, drinking herbal tea, and massage for coping with pain. In a study similar to this study, more than half of the nursing students utilize traditional and complementary medicine methods such as massage and taking warm showers, whereas more than a quarter benefit from drinking herbal tea and exercising to reduce pain [7]. In another study, nursing students use hot applications, herbal medicine, massage, and sleeping for pain management [12]. The methods most frequently utilized by the nursing students in this study are parallel to the results of studies in the literature.

The independent effects of different predictors on the PBQ score were tested with linear regression analysis using a multivariate linear regression model. According to the linear regression analysis results, when a multivariate linear regression model was established, it was found that gender, utilizing non-pharmacological interventions, and NRS scores affected the PBQ-P scores by 87.1% (R^2^=0.871). It was determined that a similar situation was also valid for the PBQ-O score (R^2^=0.810). Finally, it was observed that as the pain intensity of nursing students increased, PBQ-P (β=0.850, p<0.001) and PBQ-O (β=0.819, p<0.001) scores also increased. In a study similar to this study, the subscales of the PBQ were found to be affected by pain intensity (p<0.05) [5]. In a study conducted with liver transplant patients, a positive, moderate, statistically significant relationship was detected between both PBQ-P and NRS scores (r=0.597, p<0.05) and PBQ-O and NRS scores (r=0.471, p<0.05) [22].

The limitations that reduce the generalizability of the study include collecting data from a single center, participants' different cultural backgrounds also affecting their pain experiences, and obtaining data from participants through self-assessment. Reaching 80% of the population increased the generalizability of the results.

## Conclusions

Nursing students, the nurses of tomorrow, should be aware of the impact of psychological and organic beliefs on individuals' pain experiences and coping approaches and should take this into account when planning nursing care. As can be seen from the results of this study, the higher psychological beliefs of nursing students who use non-pharmacological interventions to cope with pain are an example of this situation. It is important to organize the nursing education content to include the evaluation of the pain experienced by individuals, the approaches they use to cope with pain, and the factors that may affect them, such as pain beliefs and pain intensity. In light of the information in this study, it should be taken into consideration that both psychological and organic beliefs have a strong relationship with pain intensity and pain coping approaches. It is believed that nursing students, who use non-pharmacological interventions as well as pharmacological methods to cope effectively with their pain, can be more tolerant toward the pain-related beliefs of individuals experiencing pain and can provide care by adopting a non-judgmental and more understanding attitude when they start working as health care professionals.
